# The relationship between perfectionism, generalized problematic internet use and bulimic behaviours

**DOI:** 10.1192/j.eurpsy.2021.332

**Published:** 2021-08-13

**Authors:** M. Pereira, A.T. Pereira, A. Araujo, M.J. Soares, J. Sobral, D. Mota, B. Maia, A. Macedo

**Affiliations:** 1 Faculty Of Medicine, University of Coimbra, Coimbra, Portugal; 2 Institute Of Psychological Medicine, Faculty Of Medicine, University of Coimbra, coimbra, Portugal; 3 Cri Psiquiatria, Centro Hospitalar Universitário de Coimbra, Coimbra, Portugal; 4 Institute Of Psychological Medicine, Faculty Of Medicine, University of Coimbra, Coimbra, Portugal

**Keywords:** Perfectionism, Generalized problematic internet use, Bulimic Behaviours

## Abstract

**Introduction:**

Perfectionism is a consistent risk factor for various psychopathological conditions, including psychological distress and eating disorders. Recently, we have shown, for the first time, that there is a relationship between perfectionism and generalized problematic internet use/GPIU (Sobral et al. 2020). Specifically, we found that the role of perfectionism in psychological disorder is partially mediated by GPIU. On the other hand, it has been suggested that the widespread use of digital media can lead to negative body image perception and abnormal eating attitudes and behaviors.

**Objectives:**

To explore, for the first time, the relationship between perfectionism, GPIU and disordered eating behavior.

**Methods:**

475 university students (78.9% girls; mean age 20.22±1.695) answered the Portuguese validated versions of: Composite Multidimensional Perfectionism Scale, GPIU Scale and Eating Attitudes Test-25. SPSS and Hayes’ Process Macro (2020) were used.

**Results:**

Bulimic Behaviours/BB significantly and moderately correlated with Perfectionist efforts (r=.263), Perfectionist concerns (r=.284) and GPIU (r=.25) (all p<.001). The mediation analyses revealed that GPIU is a partial mediator of the relationship between both perfectionism dimensions and BB.

**Conclusions:**

The evidence that both negative and “positive” perfectionism dimensions are associated to eating pathology is in line with our previous research. The present study adds, for the first time, that one of the perfectionism pathways of influence on BB operates through UGPI. Assessment and intervention to diminish eating psychopathology should focus on perfectionism and compulsive traits which could be involved in both ED and GPIU and in their comorbidity.

**Disclosure:**

No significant relationships.

